# Understanding the consumer-citizen gap in Chinese public attitudes toward farm animal welfare

**DOI:** 10.1038/s41598-024-57280-y

**Published:** 2024-03-18

**Authors:** Bing Jiang, Lihang Cui, Xiaoshang Deng, Hongbo Chen, Wenjie Tang

**Affiliations:** 1 College of Economics and Management, Northeast Agricultural University, Changjiang Street 600#, Xiangfang District, Harbin, 150030 Heilongjiang China; 2 Development Research Center of Modern Agriculture, Northeast Agricultural University, Harbin, Heilongjiang China

**Keywords:** Chinese, Public attitudes, Farm animal welfare, Consumer-citizen gaps, Consumer, Citizen, Psychology, Zoology, Environmental social sciences

## Abstract

Individuals of the general public can perform both consumer and citizen roles in farm animal welfare, and attitudes toward farm animal welfare may differ between these roles. However, scant research is available regarding this distinction, especially in developing countries such as China. The present study aimed to explore consumer-citizen gaps in Chinese public attitudes toward farm animal welfare across three dimensions and across demographic characteristics. A 36-item scale was designed, and completed by 5284 Chinese participants in a large-scale cross-sectional survey. Consumer-citizen gaps in attitudes toward farm animal welfare across three dimensions and demographic characteristics were analyzed using the Wilcoxon signed-rank test, and effects of demographic characteristics on attitudes were further explored by linear regression analysis. A significant consumer-citizen gap was found in overall attitudes, although the consumer role was only slightly more positive than the citizen role. The consumer-citizen gap is driven by differences in both cognitive attitudes and behavioral attitudes. The gap is most pronounced in cognitive attitudes, where the consumer role is significantly more positive, and smaller in behavioral attitudes, where the citizen role is significantly more positive. The consumer-citizen gap varies significantly among different demographic groups, including gender, age, education, monthly household income, area of residence, and occupation. Additionally, education, monthly household income, and area of residence have significant effects on attitudes in the dual role, whereas gender only affect consumer-role attitudes significantly. The findings provide evidence that consumer-citizen gaps in Chinese public attitudes toward farm animal welfare exist, and this distinction is mainly determined by demographic characteristics.

## Introduction

With the increasing demand for animal products, intensive livestock and poultry farming practices have seen rapid development in China^1^. While intensification has undoubtedly boosted productivity and economic returns, it has also given rise to farm animal welfare issues, thereby challenging the sustainability of animal husbandry^2^. Regrettably, farm animal welfare concerns have been largely neglected in China as the government places a strong focus on economic development^3^. This is evidenced by the lack of comprehensive nationwide legislation dedicated to farm animal welfare^4,5^.

However, recent developments point to positive changes in farm animal welfare in China. The COVID-19 pandemic has largely influenced public attitudes toward the human-animal relationship, leading to a renewed interest in animal protection and welfare within China^6^. The intricate connections between animal welfare and human health are now receiving heightened attention and fostering more extensive discourse within Chinese society^7^. The call to improve farm animal welfare has grown louder, because of its direct link to food quality^4^. In response to the demands of the public, the first national standard addressing farm animal welfare was released in 2022, which defines the criteria of farm animal welfare in culling for disease control purposes^8^. A growing number of Chinese livestock and poultry enterprises are introducing welfare-friendly animal products, particularly eggs and chicken, which have received certification from organizations like Humane Farm Animal Care, a private nonprofit organization dedicated to establishing welfare standards and certification processes for farm animals commonly raised for their products^9^. Nonetheless, the development of animal welfare in China remains in its early stages, and further practical measures are essential to promote its advancement^10^.

“Attitude” is commonly defined as an individual’s holistic subjective assessment of a particular object or subject^11^. The Theory of Planned Behavior, a widely recognized model for explaining human behavior, posits that attitudes, subjective norms, and perceived behavioral control jointly influence an individual’s behavioral intentions and actions^11^. Within this framework, attitudes are generally acknowledged as the most immediate predictors of behavior, followed by social norms and perceived behavioral control^12^. In the context of farm animal welfare, public attitudes are recognized as a potent driving force for its improvement^13^. For instance, in Europe, the mounting public concern for farm animal welfare has catalyzed the implementation of new and stricter legal provisions within the European Union^14^. Given that farm animal welfare in China is still in its early stages, understanding public attitudes toward farm animal welfare serves as a fundamental step toward improving it^10^.

Chinese public attitudes toward farm animal welfare have garnered increasing scholarly interest. There is evidence that the Chinese public self-reports a willingness to pay a premium for welfare-friendly animal products, even in cases where they may not be well-acquainted with the concept of animal welfare^4,10^. These attitudes have been found to be associated with a range of demographic characteristics, including gender, age, education, occupation, monthly household income, and area of residence^4,10,13,15,16^. Nevertheless, it is worth noting that compared to regions where farm animal welfare is more advanced, such as Europe, Oceania, and North America, there is a relative scarcity of research on this topic in the Chinese context^17^.

The fundamental economic attributes of farm animal welfare draw from the characteristics of both private goods and public goods^18^. In other words, farm animal welfare is not just a crucial quality attribute of animal products but also mirrors human conscience and ethical norms^19,20^. Consequently, an individual usually play dual roles in the relationship with farm animal welfare: one as consumer role of animal products and the other as general citizen role^21,22^. The role an individual plays depends on the context they are in^23^. In the consumer role, the public perceives farm animal welfare as an attribute directly linked to product quality assurance^24^. They derive private benefits from purchasing animal products associated with farm animal welfare attributes^18^. This consumer role greatly influences the market dynamics by exerting market functions, as their purchasing decisions directly impact the marketing and sale of animal products, which represent the primary revenue source for producers^25^. Conversely, in their roles as citizens, the public expresses moral and ethical concerns about farm animal welfare^26^. The improvement of farm animal welfare yields broader public benefits, such as reductions in environmental pollution and disease transmission, as well as the preservation of ecosystems^2^. Their attitudes toward farm animal welfare can exert influence on legislation and the formulation of policies and standards by reflecting the prevailing public concerns^22^. It is reasonable to argue that positive attitudes toward farm animal welfare in the dual roles have the potential to facilitate the attainment of higher levels of farm animal welfare.

Broader international research has indicated that individuals often hold varying attitudes toward the food system when playing dual roles as consumers and citizens^23,27^. For instance, people may hold views about the forms of meat production in their citizen roles, but these attitudes may be only weakly reflected in their behavior in consumer roles^21,28^. The distinction between the attitudes of individuals in the roles of consumer and citizen is referred to as the consumer-citizen gap^29,30^. Interestingly, farm animal welfare does not appear to be an exception to this phenomenon^22,31–34^. People may be critical of inhumane animal production practices or support welfare-friendly systems, but still buy the products with lower welfare standards, even though welfare-friendly products are also available^28,33^. This statement has been confirmed by several international investigation^35–37^. For example, Miele^38^ reported that while 73% of citizens expressed a general interest in animal welfare, only 39% considered animal welfare when purchasing meat, as revealed in a survey conducted in the UK and six other countries. Given that welfare-friendly animal products are currently in the generalization stage in China, their market share remains relatively low, and the number of consumers who have purchased such products is rather limited. Therefore, the consumer-citizen gap in this study focuses on the distinction between individuals’ attitudes toward farm animal welfare in their consumer and citizen roles.

In fact, the distinctive concerns may explain the consumer-citizen gap. In their roles as consumers, individuals often prioritize specific other attributes of the animal product itself, such as price, freshness, nutritional value, appearance, and taste^39,40^. In contrast, when acting as citizen roles, individuals demonstrate a heightened concern for the treatment of farm animals. They tend to approach socially sensitive subjects like animal welfare in a manner that aligns with perceived social norms^28,41^. Some scholars have also proposed that the consumer-citizen gap reflects the contrast between consumer roles’ hedonic desires and citizen roles’ responsible intentions^42^. However, prior studies have not adequately addressed this gap in the context of the Chinese public. A thorough understanding of the public’s attitudes toward farm animal welfare in their roles as citizens can help identify potential challenges in suggesting, formulating, and implementing farm animal welfare policies. Additionally, comprehending their attitudes in consumer roles can support welfare-friendly animal producers in meeting market demands. Therefore, there is a pressing need to gain a more nuanced understanding of the consumer-citizen gaps in attitudes toward farm animal welfare among the Chinese public.

In previous research on China, attitudes toward farm animal welfare were conventionally examined as a unified concept^4,10,15,16^. However, it is widely acknowledged that attitude is a multi-dimensional construct, typically classified into three distinct components: affective, cognitive, and behavioral^43,44^. This categorization has given rise to the three-dimensional model of attitude^45,46^. The affective component refers to the emotional evaluation of an object and reflects the emotional underpinnings associated with it. The cognitive component involves the mental conceptualization of the object, encompassing thoughts and beliefs about it. The behavioral component relates to actions directed toward the object, such as intended behaviors^47–49^. The adoption of the three-dimensional model of attitude offers a vital conceptual framework for assessing attitudes across these three dimensions. Utilizing these dimensions can help researchers obtain a comprehensive understanding of the various facets of attitude. To gain a comprehensive insight into Chinese public attitudes toward farm animal welfare, it is imperative to investigate, measure, and analyze attitudes from all three dimensions.

In this study, the attitudes of the Chinese public in their dual roles toward farm animal welfare were investigated and evaluated across three key dimensions (affective attitude, cognitive attitude, and behavioral attitude). A comparative analysis of consumer-citizen gaps in attitudes was conducted across three dimensions and various demographic characteristics such as gender, age, education, occupation, monthly household income, and area of residence. Moreover, this study considered these demographic characteristics as potential predictors of attitudes toward farm animal welfare, and explored the consumer-citizen gaps in the impact of demographic characteristics on dual role attitudes. The findings of this research provide valuable insights into Chinese public attitudes toward farm animal welfare.

## Materials and methods

This study was conducted according to the guidelines of the Declaration of Helsinki and was approved by the Ethics Committee of Northeast Agricultural University (Ethics approval code: NEAUEC20230607).

### Questionnaire

The consumer-citizen gap in attitudes toward farm animal welfare can be identified through general attitude theory and the discrepancies between measured attitudes in the consumer and citizen roles^28^. Therefore, a structured questionnaire was designed for this study. The questionnaire consisted of three main sections. In the first section, participants were provided with a text explaining the Five Freedoms. Research has shown that the majority of the Chinese public had limited prior knowledge of animal welfare^4,10^. In such cases, participants’ subjective responses obtained through direct interviews may lack reliability. Therefore, to ensure the reliability of responses, participants were given an initial understanding of farm animal welfare using the Five Freedoms text. The Five Freedoms can describe the broader dimensions of farm animal welfare for lay audiences rather than providing specific definitions, which may not lead to significant social desirability bias^3,50^. The specific text was described as follows: “Farm animal welfare describe society’s expectations for the conditions animals should experience when under human control, namely Freedom from hunger, malnutrition and thirst; Freedom from fear and distress; Freedom from heat stress or physical discomfort; Freedom from pain, injury and disease; Freedom to express normal patterns of behavior”^51^.

The second section included 36 items related to attitudes toward farm animal welfare based on the three-dimensional model of attitude, which were adapted from well-established scales used in previous studies^4,10,15,52–63^. However, in many cases, the scales used in previous research have tended to focus exclusively on attitudes in a single role, failing to account for the attitudes of individuals acting simultaneously in both consumer and citizen roles and the differences between them. Therefore, it was essential to identify and categorize items from existing research to examine attitudes toward farm animal welfare in the corresponding role. The classification of items was according to the roles they address and the definition of the three dimensions of attitude. All items in the questionnaire utilized a 5-point Likert-type response format. Participants were asked to rate their agreement with each statement on a scale ranging from 1 (strongly disagree) to 5 (strongly agree). Higher scores indicated more positive attitudes toward farm animal welfare. The original English version of the items was translated into Chinese and back-translated into English to ensure accuracy and consistency. To ensure the quality of the questionnaire, five experts were asked to review the items and a pilot survey of 318 respondents was carried out via the Internet before the formal survey. Then, any unclear or ambiguous questions were modified and corrected to improve clarity without changing the essence of the questions. To minimize potential ordering effects, the order of the 36 items was randomized for each participant. The detailed statements and references of the final items are provided in Table 1.

**Table 1:** Questionnaire items on attitudes toward farm animal welfare.

Dual role	Statement	References
Consumer role	I am concerned about the welfare of farm animals raised for human consumption	^52^
	I feel compassion for farm animals raised for human consumption	^53^
	I believe the use of violence on farm animals raised for human consumption is morally reprehensible	^54^
	I believe that farm animals raised for human consumption should be treated with dignity	^52^
	Ensuring the proper care of farm animals raised for human consumption is important to me	^55^
	I believe that improving the welfare of farm animals raised for human consumption is more important than the pursuit of low prices	
Citizen role	I believe that farm animals possess physical sensations similar to those of humans	^56^
	I believe that farm animals can experience emotions similar to those of humans	
	Improving farm animal welfare brings me a sense of fulfillment	^4^
	I believe that farm animals have an inherent right to life similar to that of humans	^57^
	I believe that intensive farming practices raise serious ethical concerns regarding the treatment of farm animals	^58^
	I believe that individuals who oppose intensive farming practices are driven by genuine concern rather than being overly sentimental	^15^
Consumer role	I actively keep myself informed about issues related to the welfare of farm animals raised for human consumption	^59^
	I believe I possess ample understanding about the living conditions of farm animals raised for human consumption	
	I believe that the conditions during transport have an impact on the welfare of farm animals raised for human consumption	^54^
	I believe that pre-slaughter practices have an impact on the welfare of farm animals raised for human consumption	
	I believe that farm animals raised for human consumption perform better when they are in comfortable conditions	^59^
	I believe that improving the welfare of farm animals raised for human consumption leads to higher-quality animal products	^52^
Citizen role	I have heard of the phrase “farm animal welfare”	^4^
	I have an understanding of the concept of farm animal welfare	^10^
	I believe that improving farm animal welfare benefits both humans and animals	^60^
	I support the inclusion of farm animal welfare in school curricula	
	I believe that the current standards of farm animal welfare in China are falling behind those of developed countries	^4^
	I believe that it is important to establish legislation for farm animal welfare	^10^
Consumer role	Consideration of farm animal welfare plays a role in my choices when purchasing animal products	^61^
	I would consider changing my choice of store to purchase welfare-friendly products	^62^
	I have a preference for animal products that meet established animal welfare standards over regular products	^54^
	I am willing to pay a premium for welfare-friendly animal products	^61^
	I believe that it is difficult to identify the welfare conditions of farm animals using the information available on animal products	^54^
	I believe that welfare-friendly animal products should be officially certified and labelled	^63^
Citizen role	I treat farm animals well when I have contact with them	^54^
	I intervene when I witness farm animal abuse	
	I would like to tell people around me about farm animal welfare	
	I would like to be involved in the improvement of the welfare of farm animals	^15^
	I am open to donating to organizations dedicated to improving farm animal welfare	
	I support the implementation of legislation and the establishment of national standards to protect the welfare of farm animals	^10^

The third section collected demographic information from participants, including gender, age, education, monthly household income, area of residence, occupation, and region.

### Procedure

The data for this study were collected through a cross-sectional survey conducted from July 15, 2023, to August 9, 2023, which coincided with the school summer holiday period. The survey was carried out by a team of 25 investigators recruited from undergraduate and graduate students at the College of Economics and Management, Northeast Agricultural University. These investigators underwent standardized training and post-training assessments. Subsequently, they returned to their hometowns to conduct face-to-face interviews utilizing a structured questionnaire during the summer holiday. The survey covered 119 rural and urban areas within the jurisdiction of 25 provinces across six regions of mainland China, including North China (Beijing, Hebei, Shanxi, and Inner Mongolia), Northeast China (Liaoning, Jilin, and Heilongjiang), East China (Shanghai, Jiangsu, Zhejiang, Anhui, Fujian, Jiangxi, and Shandong), South Central China (Henan, Hubei, Hunan, Guangdong, and Guangxi), Southwest China (Sichuan, Guizhou, and Yunnan), and Northwest China (Shaanxi, Gansu, and Xinjiang). The selection of survey sites and participants adhered to the principles of random sampling. The proportion of participants interviewed in business areas (e.g., shops and supermarkets) and public areas (e.g., parks, schools, streets, and squares) was 1:1.42.

Participants are often unaware of the disparity in attitudes between their roles as citizens and consumers^42^. Therefore, to assist participants in distinguishing between their roles as citizens and consumers, situational information was provided before they responded to the items^64^. For the consumer role, the situational information was “In the role of a consumer, imagine shopping for animal products at your regular store. Please choose an answer based on this scenario and your personal feelings.” For the citizen role, the situational information was “In the role of a citizen, imagine attending a community meeting to discuss policies related to farm animal welfare. Please choose an answer based on this scenario and your personal feelings.”

Before the commencement of the survey, all participants were informed about the purpose of the survey, which focused on assessing their attitudes toward farm animal welfare. It was explicitly communicated to them that their responses would be treated with confidentiality and solely utilized for scientific research purposes. Additionally, participants were provided with an informed consent document, which outlined the conditions of their participation, as well as their right to withdraw from the survey at any stage. The survey proceeded only upon receiving explicit confirmation of consent from the participants.

### Participants

To ensure that the participants embodied both consumer and citizen roles, individuals who had never purchased or consumed farm animal products were excluded. Additionally, individuals under the age of 18 and over the age of 70 were excluded. This approach ensured that each participant possessed personal experience related to the purchase and consumption of farm animal products and had the independent capacity to express their viewpoints, emotions, and intentions.

### Data analysis

The data analysis process encompassed the following five steps: (1) Reliability and validity tests. Reliability was evaluated using Cronbach’s alpha coefficient, and validity was assessed through the Kaiser–Meyer–Olkin (KMO) measure and Bartlett’s test of sphericity. (2) Calculation of attitude scores. The attitude scores for each dimension within a specific role were calculated as the sum of the corresponding item scores. (3) Data normality test. The Kolmogorov–Smirnov test was employed to ascertain the normal distribution of the data. (4) Identification of consumer-citizen gaps in attitudes. If the data exhibited normal distribution, a parametric paired sample *t*-test was applied to identify consumer-citizen gaps in attitudes toward farm animal welfare across three dimensions and demographic characteristics. Otherwise, a non-parametric Wilcoxon signed-rank test was conducted. (5) Examination of the consumer-citizen gaps in the impact of demographic characteristics on attitudes. To further explore the differential impact of demographic characteristics on dual role attitudes, linear regression analysis was performed. Recognizing that public attitudes toward farm animal welfare may be influenced by macro-level factors in different regions of residence, such as socio-economic development and cultural practices, fixed effects of region variables were thus controlled in the linear regression analysis. Detailed descriptions of the dependent and independent variables can be found in Table 2. All statistical tests were two-sided, and statistical significance was set at *P* < 0.05. Data were analyzed using SPSS Version 24 (IBM Corp., Armonk, NY, USA).

**Table 2:** Variable definitions and assignments.

Variable classification	Variable definitions and assignments
Dependent variables	Attitude scores in the consumer role
	Attitude scores in the citizen role
	Attitude scores for the consumer-citizen gap (calculated as Attitude scores in the consumer role minus Attitude scores in the citizen role)
Independent variables	Gender: Male = 0, Female = 1
	Age: 18–20 years = 1, 21–30 years = 2, 31–40 years = 3, 41–50 years = 4, 51–60 years = 5, 61–70 years = 6
	Education: Junior middle school and below = 1, High school = 2, Junior college = 3, Undergraduate = 4, Postgraduate = 5
	Monthly household income: < US $500 = 1, US $501–1000 = 2, US $1001–1500 = 3, US $1501–2000 = 4, > US $2000 = 5
	Area of residence: Urban area = 0, Rural area = 1
	Occupation: Unemployed = 1, Student = 2, Farmer = 3, Self-employed = 4, Employed = 5, Retired = 6
Fixed effects	Region: North China = 1, Northeast China = 2, East China = 3, South Central China = 4, Southwest China = 5, Northwest China = 6

### Ethical approval

This study was performed in line with the principles of the Declaration of Helsinki. Approval was granted by the Ethics Committee of Northeast Agriculture University (Harbin, China). Informed consent was obtained from all individual participants included in the study. No identifying data were collected from the respondents.

## Results

### Reliability and validity test

The scale demonstrated satisfactory reliability with a Cronbach’s alpha exceeding 0.7. Specifically, for internal consistency, the Cronbach’s alpha coefficients were 0.714 for the entire scale, and 0.726, 0.740, and 0.739 for the affective attitude, cognitive attitude, and behavioral attitude items, respectively. The scale also exhibited good construct validity, as indicated by a KMO value greater than 0.8 and a significant result in Bartlett’s test of sphericity. Specifically, for internal construct validity, the KMO value was 0.845, and the chi-square value of Bartlett’s test of sphericity was 8601.573 (*df* = 630, *P* < 0.001), confirming its statistical significance. In summary, the scale successfully passed the reliability and validity tests, affirming its good reliability and validity.

### Sample characteristics

A total of 5537 respondents participated in the survey, with each investigator visiting a minimum of two cities (mean = 2.28) within one province and conducting interviews with at least 200 respondents on average (mean = 221.48). After excluding questionnaires with missing data and those of poor quality (*n* = 253), a total of 5284 questionnaires were collected, resulting in an effective questionnaire rate of 95.43%. Demographic characteristics of the participants and the results of the 2020 Population Census obtained from the National Bureau of Statistics are presented in Table 3.

**Table 3:** Demographic characteristics of the participants and 2020 Population Census.

Demographics	*n* = 5284 (%)	2020 Population census	
Gender	Male	2741 (51.87)	51.24%
	Female	2543 (48.13)	48.76%
Age	18–20 years	275 (5.20)	Mean = 38.8 years
	21–30 years	1714 (32.44)	
	31–40 years	1678 (31.76)	
	41–50 years	872 (16.50)	
	51–60 years	498 (9.43)	
	61–70 years	247 (4.67)	
Education	Junior middle school and below	632 (11.96)	69.44%
	High school	909 (17.20)	15.09%
	Junior college	2030 (38.42)	15.47%
	Undergraduate	1269 (24.02)	
	Postgraduate	444 (8.40)	
Monthly household income	< US $500	1023 (19.36)	mean = US $1018.83
	US $501–1000	1518 (28.73)	
	US $1001–1500	1670 (31.60)	
	US $1501–2000	707 (13.38)	
	> US $2000	366 (6.93)	
Area of residence	Urban area	3428 (64.88)	63.89%
	Rural area	1856 (35.12)	36.11%
Occupation	Unemployed	298 (5.64)	4.20%
	Students	895 (16.94)	18.64%
	Farmers	409 (7.74)	14.69%
	Self-employed	911 (17.24)	18.55%
	Employed	2582 (48.86)	34.61%
	Retired	189 (3.58)	9.31%
Region	North China	835 (15.80)	11.99%
	Northeast China	609 (11.53)	6.98%
	East China	1176 (22.26)	27.35%
	South Central China	1289 (24.39)	29.03%
	Southwest China	854 (16.16)	14.53%
	Northwest China	521 (9.86)	10.12%

Monthly household income was converted at the average exchange rate for 2020, which was CNY 6.897 per USD.

The gender ratio (male/female) of the sample was approximately 1:0.93, with 2741 male participants and 2543 female participants. The mean age of all participants was 35.75 years, with the highest percentage of participants falling within the 21–30 years age group (*n* = 1714, 32.44%), followed by those in the 31–40 years age group (*n* = 1678, 31.76%). The majority of participants held a junior college degree or higher (*n* = 3743, 70.84%), while those with a high school education or below accounted for nearly 30% (*n* = 1541, 29.16%). The monthly household income of the sample exhibited a mode and median within the range of US $1001–1500, and participants with monthly household income exceeding US $1000 constituted more than half of the sample (*n* = 2743, 51.91%). Participants living in rural areas comprised more than three in ten (*n* = 1856, 35.12%), whereas more than 60% resided in urban areas (*n* = 3428, 64.88%). Nearly half of the participants were employed (*n* = 2582, 48.86%), followed by those who were self-employed (*n* = 911, 17.24%), and students (*n* = 895, 16.94%). Nearly a quarter of the participants were from South Central China (*n* = 1289, 24.39%), with more than a fifth coming from East China (*n* = 1176, 22.26%), followed by those from Southwest China (*n* = 854, 16.16%), North China (*n* = 835, 15.80%), Northeast China (*n* = 609, 11.53%), and Northwest China (*n* = 521, 9.86%).

### Overview of public attitudes toward farm animal welfare in China

The scores for the scale are given in Table 4. With regard to affective attitudes in consumer roles, nearly half of the participants expressed concern (*n* = 2578, 48.79%) and compassion (*n* = 2353, 44.53%) for farm animals. While the majority of participants (*n* = 4161, 78.75%) believed that using violence on farm animals is morally reprehensible, only a minority *(n* = 1702, 32.31%) felt that these animals should be treated with dignity. More than 40% of the participants (*n* = 2178, 41.22%) considered proper care for farm animals to be important. However, this percentage slightly decreased (*n* = 2135, 40.40%) when compared to the importance of low prices.

**Table 4:** Responses of the scales measuring attitudes toward farm animal welfare.

Statement	Strongly disagree	Disagree	Neutral	Agree	Strongly agree
I am concerned about the welfare of farm animals raised for human consumption	354	550	1802	1504	1074
I feel compassion for farm animals raised for human consumption	546	994	1391	1294	1059
I believe the use of violence on farm animals raised for human consumption is morally reprehensible	172	359	592	1621	2540
I believe that farm animals raised for human consumption should be treated with dignity	1055	1443	1084	993	709
Ensuring the proper care of farm animals raised for human consumption is important to me	680	1186	1240	1220	958
I believe that improving the welfare of farm animals raised for human consumption is more important than the pursuit of low prices	1206	1107	836	973	1162
I believe that farm animals possess physical sensations similar to those of humans	57	177	403	1860	2787
I believe that farm animals can experience emotions similar to those of humans	84	216	416	1824	2744
Improving farm animal welfare brings me a sense of fulfillment	922	877	2331	683	471
I believe that farm animals have an inherent right to life similar to that of humans	2073	2773	346	63	29
I believe that intensive farming practices raise serious ethical concerns regarding the treatment of farm animals	347	1253	1692	1484	508
I believe that individuals who oppose intensive farming practices are driven by genuine concern rather than being overly sentimental	251	475	1510	1849	1199
I actively keep myself informed about issues related to the welfare of farm animals raised for human consumption	2635	1873	414	217	145
I believe I possess ample understanding about the living conditions of farm animals raised for human consumption	2942	1957	246	83	56
I believe that the conditions during transport have an impact on the welfare of farm animals raised for human consumption	374	627	933	2226	1124
I believe that pre-slaughter practices have an impact on the welfare of farm animals raised for human consumption	440	649	816	2359	1020
I believe that farm animals raised for human consumption perform better when they are in comfortable conditions	174	385	712	2194	1819
I believe that improving the welfare of farm animals raised for human consumption leads to higher-quality animal products	158	272	540	2229	2085
I have heard of the phrase “farm animal welfare”	2867	1151	683	379	204
I have an understanding of the concept of farm animal welfare	2943	1427	539	216	159
I believe that improving farm animal welfare benefits both humans and animals	273	521	1083	2126	1281
I support the inclusion of farm animal welfare in school curricula	323	608	2805	852	696
I believe that the current standards of farm animal welfare in China are falling behind those of developed countries	315	428	2033	1393	1115
I believe that it is important to establish legislation for farm animal welfare	317	516	956	1433	2062
Consideration of farm animal welfare plays a role in my choices when purchasing animal products	593	1415	989	1408	879
I would consider changing my choice of store to purchase welfare-friendly products	657	1724	1156	1196	551
I have a preference for animal products that meet established animal welfare standards over regular products	275	667	1099	1866	1377
I am willing to pay a premium for welfare-friendly animal products	497	996	1227	1405	1159
I believe that it is difficult to identify the welfare conditions of farm animals using the information available on animal products	131	832	1575	1776	970
I believe that welfare-friendly animal products should be officially certified and labelled	251	479	763	1987	1804
I treat farm animals well when I have contact with them	23	71	822	1792	2576
I intervene when I witness farm animal abuse	124	529	1362	1207	2062
I would like to tell people around me about farm animal welfare	613	862	1519	1363	927
I would like to be involved in the improvement of the welfare of farm animals	884	1443	1715	736	506
I am open to donating to organizations dedicated to improving farm animal welfare	1023	1511	294	1061	1395
I support the implementation of legislation and the establishment of national standards to protect the welfare of farm animals	331	601	924	1446	1982

In terms of affective attitudes in citizen roles, the majority of participants believed that farm animals have both physical sensations (*n* = 4647, 87.94%) and emotions (*n* = 4568, 86.45%). Nearly half of the participants (*n* = 2331, 44.11%) expressed neutrality regarding the connection between their personal fulfillment and the improvement of farm animal welfare. Over 90% of the participants (*n* = 4846, 91.71%) strongly disagreed or disagreed with the statement that farm animals have the same right to life as humans. Almost 40% of the participants (*n* = 1992, 37.70%) believed that intensive farming practices raise serious ethical concerns. Nearly 60% believed (*n* = 3048, 57.68%) that those who oppose intensive farming practices are justified.

Regarding cognitive attitudes in consumer roles, a relatively small number of participants (*n* = 362, 6.85%) were actively informed about farm animal welfare issues, and even fewer (*n* = 139, 2.63%) believed they possessed sufficient understanding of farm animal living conditions. Over 60% of the participants recognized that transportation (*n* = 3350, 63.40%) and pre-slaughter practices (*n* = 3379, 63.95%) have impacts on farm animal welfare. The majority of participants were aware of the positive effects of improving farm animal welfare on performance and products quality, with 75.95% (*n* = 4013) and 87.32% (*n* = 4314) strongly agreeing or agreeing with the statements, respectively.

In relation to cognitive attitudes in citizen roles, a majority of the participants had not heard of the phrase “farm animal welfare” (*n* = 4018, 76.04%) and were unfamiliar with its concept (*n* = 4370, 82.70%). Although nearly two-thirds of the participants (*n* = 3407, 64.48%) strongly agreed or agreed on the benefits of improving farm animal welfare for both humans and animals, most (*n* = 2805, 53.08%) had no strong opinion on whether it should be included in school curricula. Nearly 50% of the participants (*n* = 2508, 47.46%) believed that farm animal welfare standards in China lagged behind those in developed countries, and a larger number (*n* = 3495, 66.14%) emphasized the importance of legislation for farm animal welfare.

With respect for behavioral attitudes in consumer roles, 43.28% of the participants (*n* = 2287) believed that farm animal welfare influenced their choices of animal products, but only 33.06% (*n* = 1747) were willing to switch stores to purchase welfare-friendly products. While over 60% of the participants (*n* = 3243, 61.37%) preferred welfare-friendly products, fewer than 50% (*n* = 2564, 48.52%) were willing to pay a premium for them. Over 50% of the participants (*n* = 2746, 51.97%) found it challenging to identify the welfare status of products based on available information, and more than 70% (*n* = 3791, 71.74%) supported the official certification and labeling of welfare-friendly animal products.

Concerning behavioral attitudes in citizen roles, the vast majority of participants (*n* = 4368, 82.66%) reported treating farm animals well, and more than 60% (*n* = 3269, 61.87%) expressed their willingness to intervene in cases of farm animal cruelty. Over 40% of the participants (*n* = 2290, 43.34%) were willing to inform others about farm animal welfare, but only slightly more than 20% (*n* = 1242, 23.50%) were inclined to engage in substantive efforts. Regarding donations to farm animal welfare organizations, the responses from participants were polarized, with 47.96% of them (*n* = 2534) being unwilling and 46.48% (*n* = 2456) willing to contribute. As for legislation and national standards to protect farm animal welfare, 37.51% of the participants (*n* = 1982) strongly agreed, and 27.37% agreed (*n* = 1446).

### Consumer-citizen gaps in attitudes toward farm animal welfare across three dimensions

Based on the results of the Kolmogorov–Smirnov normality test, it was evident that the assumption of normal distribution was not satisfied (*P* < 0.001). Therefore, as all the data exhibited a non-normal distribution, a non-parametric Wilcoxon signed-rank test was employed to identify differences in attitudes toward farm animal welfare between the consumer role and the citizen role.

The results of the comparison of attitudes toward farm animal welfare in dual roles across three dimensions are presented in Table 5. Across three dimensions, the participants scored the highest in behavioral attitudes, followed by affective attitudes, and exhibited the lowest scores in cognitive attitudes.

**Table 5:** Results of comparing the dual role attitudes across three dimensions.

Dimension	Role	Mean ± *SD*	Median	Comparison
Overall	Consumer	58.49 ± 4.871	59.00	*Z* = − 3.382, *P* = 0.001
	Citizen	58.47 ± 4.573	59.00	
Affective component	Consumer	19.69 ± 3.365	20.00	*Z* = − 0.396, *P* = 0.692
	Citizen	19.89 ± 2.266	20.00	
Cognitive component	Consumer	18.49 ± 2.532	19.00	*Z* = − 9.163, *P* < 0.001
	Citizen	17.64 ± 2.547	18.00	
Behavioral component	Consumer	20.31 ± 2.836	20.00	*Z* = − 10.683, *P* < 0.001
	Citizen	20.93 ± 2.988	21.00	

Regarding overall attitudes, 47.16% of the participants (*n* = 2492) demonstrated higher scores in the citizen role, while 46.23% (*n* = 2443) displayed higher scores in the consumer role. The participants exhibited significantly more positive overall attitudes toward farm animal welfare in the consumer role than in the citizen role.

In terms of affective attitudes, 43.17% of the participants (*n* = 2281) scored higher in the consumer role than in the citizen role, while 47.48% (*n* = 2509) had the opposite result. The participants’ citizen role displayed more positive affective attitudes toward farm animal welfare compared to the consumer role, but the difference was not found to be statistically significant.

Concerning cognitive attitudes, nearly 60% of the participants (*n* = 3067, 58.04%) achieved higher scores in the consumer role, while more than 30% (*n* = 1618, 30.62%) scored higher in the citizen role. Cognitive attitudes toward farm animal welfare of the participants in the consumer role were significantly more positive than those in the citizen role.

With respect to behavioral attitudes, more than half of the participants (*n* = 2706, 51.21%) achieved higher scores in the citizen role, while nearly 40% (*n* = 2086, 39.48%) scored higher in the consumer role. More positive behavioral attitudes toward farm animal welfare were observed in the participants’ citizen role compared with the consumer role, and the Wilcoxon signed-rank test confirmed that the difference was significant.

### Consumer-citizen gaps in attitudes toward farm animal welfare across demographic characteristics

The results of the comparison of attitudes toward farm animal welfare in dual roles across various demographic characteristics are presented in Table 6. For gender, male participants exhibited significantly more positive attitudes in the citizen role compared to the consumer role. In contrast, female participants displayed significantly more positive attitudes in the consumer role compared to the citizen role.

**Table 6:** Results of comparing dual role attitudes across demographic characteristics.

Demographics	Consumer role		Citizen role		Comparison
	*M* ± *SD*	Median	*M* ± *SD*	Median	
Male	52.52 ± 4.880	52.00	55.37 ± 4.623	55.00	*Z* = − 3.396, *P* < 0.001
Female	64.93 ± 4.863	65.00	61.80 ± 4.518	62.00	*Z* = − 4.753, *P* < 0.001
18–20 years	60.30 ± 4.219	61.00	61.05 ± 3.927	61.00	*Z* = − 1.147, *P* = 0.147
21–30 years	58.71 ± 4.678	59.00	59.08 ± 4.500	59.00	*Z* = − 3.171, *P* = 0.002
31–40 years	57.43 ± 5.059	58.00	57.61 ± 4.742	58.00	*Z* = − 3.967, *P* < 0.001
41–50 years	58.35 ± 5.006	58.00	57.52 ± 4.776	58.00	*Z* = − 3.314, *P* = 0.002
51–60 years	58.98 ± 4.845	59.00	58.54 ± 4.278	59.00	*Z* = − 1.400, *P* = 0.001
61–70 years	61.69 ± 4.729	62.00	60.32 ± 4.325	60.00	*Z* = − 3.242, *P* = 0.002
Junior middle school and below	57.72 ± 4.454	58.00	56.73 ± 4.083	57.00	*Z* = − 3.923, *P* < 0.001
High school	57.95 ± 4.922	58.00	57.46 ± 4.908	58.00	*Z* = − 2.549, *P* = 0.011
Junior college	58.69 ± 4.714	59.00	58.60 ± 4.439	59.00	*Z* = − 2.249, *P* = 0.032
Undergraduate	58.81 ± 5.259	58.00	58.64 ± 4.935	59.00	*Z* = − 3.917, *P* < 0.001
Postgraduate	58.89 ± 4.438	60.00	61.88 ± 3.698	61.00	*Z* = − 2.981, *P* = 0.003
< US $500	54.71 ± 5.144	55.00	55.31 ± 5.564	56.00	*Z* = − 6.583, *P* < 0.001
US $501–1000	55.85 ± 4.675	56.00	56.53 ± 4.655	57.00	*Z* = − 7.556, *P* < 0.001
US $1001–1500	57.31 ± 4.354	57.00	58.66 ± 4.205	59.00	*Z* = − 4.093, *P* < 0.001
US $1501–2000	64.22 ± 4.432	64.00	61.12 ± 3.687	61.00	*Z* = − 4.691, *P* < 0.001
> US $2000	74.36 ± 4.516	74.00	69.29 ± 3.659	69.00	*Z* = − 4.845, *P* < 0.001
Urban area	59.17 ± 5.135	59.00	60.48 ± 4.968	60.00	*Z* = − 2.979, *P* = 0.003
Rural area	57.24 ± 4.344	57.00	54.74 ± 3.677	55.00	*Z* = − 3.865, *P* < 0.001
Unemployed	53.04 ± 4.153	53.00	55.86 ± 3.532	56.00	*Z* = − 8.009, *P* < 0.001
Student	60.30 ± 4.904	60.00	63.34 ± 4.589	63.00	*Z* = − 5.964, *P* < 0.001
Farmer	53.89 ± 4.050	54.00	52.88 ± 4.064	53.00	*Z* = − 3.343, *P* = 0.001
Self-employed	59.23 ± 4.428	59.00	57.54 ± 4.056	58.00	*Z* = − 4.318, *P* < 0.001
Employed	58.55 ± 5.171	59.00	58.03 ± 4.985	58.00	*Z* = − 3.956, *P* < 0.001
Retired	64.15 ± 5.197	64.00	61.98 ± 5.965	62.00	*Z* = − 3.181, *P* = 0.001

Regarding age, more positive attitudes toward farm animal welfare in the consumer role were observed within the age groups of 18–40 years, while the opposite trend was observed within the age groups of 41–70 years. Statistical significance was achieved in the differences of participants’ dual role attitudes within the age groups of 21–30 years, 31–40 years, 41–50 years, 51–60 years, and 61–70 years. However, the differences in dual role attitudes did not reach statistical significance for the age groups of 18–20 years.

In terms of education, participants with postgraduate degrees had more positive attitudes in the citizen role than in the consumer role, while the reverse was observed among participants with undergraduate degrees and below, who demonstrated more positive consumer role attitudes. The differences in dual role attitudes toward farm animal welfare were statistically significant for all participants with educational backgrounds ranging from junior middle school and below to postgraduate.

With regard to monthly household income, participants with monthly household incomes below US $500, US $501–1000, and US $1001–1500 displayed more positive citizen role attitudes toward farm animal welfare compared to the consumer role, and the differences were statistically significant. Conversely, participants with monthly household incomes of US $1501–2000 and over US $1500 showed significantly more positive attitudes in the consumer role than in the citizen role.

Concerning the area of residence, statistically significant differences were found in dual role attitudes toward farm animal welfare between participants living in urban areas and those in rural areas. However, these differences were in opposite directions, with participants living in urban areas exhibiting more positive citizen role attitudes, while those living in rural areas displayed more positive consumer role attitudes.

With respect to occupation, for participants who were unemployed and students, attitudes were significantly more positive in the citizen role. The remaining participants who were farmers, self-employed, employed, and retired, had significantly more positive attitudes in the consumer role.

### The impact of demographic characteristics on attitudes toward farm animal welfare

The collinearity test results showed a maximum VIF of 3.52 and a mean VIF of 2.05, with all VIF values for the independent variables below 5, indicating the absence of multicollinearity. The dependent variables in Model 1 and Model 2 were the attitude scores in the consumer role, the attitude scores in the citizen role, and the attitude scores for the consumer-citizen gap, respectively. The results of the *F*-test suggested that Model 1 (*F*_(11, 5272)_ = 66.65, *P* < 0.001) and Model 2 (*F*_(11, 5272)_ = 48.11, *P* < 0.001) were statistically significant overall. Goodness-of-fit statistics further indicated an overall good fit for Model 1 (*R*^*2*^ = 0.9221, Adj *R*^2^ = 0.9203) and Model 2 (*R*^2^ = 0.8364, Adj* R*^2^ = 0.8344). Detailed results of the linear regression are presented in Table 7.

**Table 7:** Results of the linear regression analysis.

Variable	Model 1 (Consumer role)				Model 2 (Citizen role)			
	***B***	***SE***	***t***	***P***	***B***	***SE***	***t***	***P***
Gender	6.986***	0.529	13.21	< 0.001	0.980	0.524	1.87	0.061
Age	− 0.264	0.554	− 0.48	0.633	0.600	0.544	1.10	0.270
Education	5.002***	0.296	16.92	< 0.001	3.408***	0.291	11.71	< 0.001
Monthly household income	3.920***	0.192	20.42	< 0.001	2.318***	0.191	12.14	< 0.001
Area of residence	− 2.240***	0.217	− 10.34	< 0.001	− 1.408***	0.216	− 6.52	< 0.001
Occupation	0.446	0.320	1.39	0.164	0.260	0.318	0.82	0.430
Constant	46.481***	0.693	67.07	< 0.001	50.662***	0.682	74.28	< 0.001

*P < 0.05, **P < 0.01, and ***P < 0.001.

In Model 1, gender exhibited a significant and positive influence on consumer role attitudes, indicating that female participants had more positive attitudes in their consumer role compared to male participants. Education demonstrated a significant positive impact on consumer role attitudes, revealing a direct relationship between higher levels of education and more positive attitudes in the consumer role. Monthly household income was found to have a significant and positive effect on consumer role attitudes, indicating that higher monthly household income among participants was associated with more positive consumer attitudes. The negative interaction between the area of residence and consumer role attitudes was statistically significant, suggesting that participants residing in urban areas tended to have more positive attitudes in their consumer role than those living in rural areas. Age and occupation did not have statistically significant effects on consumer role attitudes toward farm animal welfare.

In Model 2, similar to Model 1, education and monthly household income were significantly and positively associated with citizen role attitudes, and the area of residence had a significant negative impact on citizen role attitudes. In contrast to Model 2, no significant relationship was observed between gender and citizen role attitudes. Additionally, there were no statistically significant effects of age and occupation on citizen role attitudes were observed.

## Discussion

Given the increasing public concern about farm animal welfare in China, a comprehensive understanding of Chinese public attitudes toward this topic is crucial for informing the development and improvement of farm animal welfare. However, there is a scarcity of research on the consumer-citizen gaps in attitudes toward farm animal welfare among the Chinese public. Therefore, the present study aimed to assess and analysis Chinese public attitudes toward farm animal welfare across three dimensions and demographic characteristics.

Before further discussion of the results, a discussion of the sample distribution is necessary. The gender distribution and area of residence in the sample were closed to the results of the 2020 Population Census. However, the sample displayed a younger demographic, higher educational attainment, and a greater average monthly household income. The proportions of participants in each occupation category and their distribution across different regions showed slight discrepancies compared to the 2020 Population Census results. This divergence is primarily attributed to the fact that the 2020 Population Census encompassed all Chinese citizens, but not all of whom had consumer roles, such as vegetarians. Furthermore, the 2020 Population Census included individuals who were excluded from this study, such as citizens under 18 and those over 70 years old. As a result, the sample in this study is considered to be comparatively representative and comprehensive.

### Features of Chinese public attitudes toward farm animal welfare

Given that the average attitude scale score of the participants (Mean = 58.48 ± 4.724) exceeded the neutral midpoint (54.00), this study reinforces the findings from earlier research that the majority of the Chinese public hold positive attitudes toward farm animal welfare^4,10,13,15^. To some extent, the participants’ responses could reflect the features of Chinese public attitudes toward farm animal welfare.

The participants’ responses regarding affective attitudes suggest that the participants exhibit care and empathy toward farm animals from an ethical and moral standpoint. However, this compassion is conservative, rather than radical, which primarily focused on animal welfare considerations and does not extend to the realm of animal rights. In fact, there is still a large portion of the Chinese public believes that animal welfare is premature for Chinese society^65^. They argue that human welfare and human issues have not been adequately addressed in China, and there are insufficient laws in place for the protection of human rights^66^.

The participants’ responses regarding cognitive attitudes indicate that they recognized the benefits of improving farm animal welfare, but they demonstrated limited knowledge about this subject. Similar results have been found in several studies, emphasizing that public concern for farm animal welfare in China is largely driven by its positive impact on food quality and safety^3,4^. Information about animal welfare is scarce among the Chinese public due to limited coverage in official media and school curricula^10^. The rise of social media in recent years has ignited increased public interest in animal welfare. However, animal welfare remains a relatively new concept in China, various levels of doubt, resistance, or confusion still persist among the public regarding animal welfare^65,66^. Therefore, future priority is to facilitate a comprehensive public understanding of animal welfare through publicity and promotional initiatives^67^.

Regarding behavioral attitudes, the participants tend to be free-riders when it comes to improving farm animal welfare. The free-rider behaviors were equally present in other countries, such as America^68^ and Germany^69^. In principle, this potential free-rider problem could lead to unsustainable improvements in farm animal welfare if the cost that the public is willing to bear is less than the amount required for improving farm animal welfare^70^. Therefore, it is imperative for the Chinese government to address the potential free-rider problem by actively responding to public calls for farm animal welfare standards, legislation, certification, and labeling.

### Consumer-citizen gaps in attitudes across three dimensions

The results show that the overall attitudes of the consumer role were slightly more positive than those of the citizen role. The present study confirms previous research findings, which indicated the existence of consumer-citizen gaps in Chinese public attitudes toward farm animal welfare^22,31–34^.

With regard to the affective component, the results suggest that there was no significant difference in scores between the participants in their consumer role and citizen role. The public’s affective attitudes toward farm animal welfare are rooted in a fundamental moral concern for the welfare of farm animals^71^. While the dual roles may hold different concerns and specific viewpoints on certain issues, the influence of common social and cultural factors fosters a shared moral value^15,72^. This moral value is consistent across both consumer and citizen roles, as they both share the belief in the importance of treating farm animals well^33^. This common value contributes to the development of similar affective attitudes across these two roles.

In terms of the cognitive component, the results indicate that participants scored significantly higher in the consumer role compared to the citizen role. Rapid urbanization in China has led to increased social and spatial distance between the general public and the livestock sector, which has resulted in limited knowledge about livestock and poultry farming practices and farm animal welfare among the public^31,73,74^. However, as the purchase choices of livestock products directly impact the self-interest of the consumer role, they are typically more motivated to comprehend the attribute of farm animal welfare through means such as reading product labels and merchant advocacy^75–77^. In contrast, the citizen role is more likely to acquire information about animal welfare through news media and social media^78^. This information often emphasizes negative aspects, such as incidents of animal cruelty, and has a relatively lesser impact on the growth of knowledge^79,80^. Consequently, the consumer role gains more knowledge and experience about farm animal welfare compared to the citizen role.

Regarding the behavioral component, the results reveal that participants scored significantly higher in the citizen role than in the consumer role. This may be attributed to the preference of the consumer role. Previous studies have shown that Chinese consumers prioritize other attributes of animal products, such as their origin, quality, organic certification, appearance, and traceability, over farm animal welfare^81–83^. This differs from consumers in countries where animal welfare is more advanced^84^. Farm animal welfare is a credence attribute, and welfare-friendly animal products are considered credence goods^85,86^. The asymmetrically distributed information about food credence attributes, such as animal welfare, can diminish consumer trust and, consequently, reduce their willingness to pay for such attributes^87,88^. This phenomenon may be especially pronounced in China, where farm animal welfare is still in its early stages of development, as welfare-friendly animal products have not gained widespread popularity and there is a lack of a comprehensive certification and labeling system for such products.

### Consumer-citizen gaps in attitudes across demographic characteristics and the impact of demographic characteristics on dual role attitudes

Female participants displayed significantly more positive attitudes in the consumer role compared to the citizen role, whereas the opposite pattern was observed for male participants. This can be attributed to the fact that women are typically the primary food shopper in their families and pay more attention on food quality, the health of family members, and the attributes of animal products^34,62^. This result is further supported by the significant positive impact of gender on attitudes in the consumer role, which is consistent with previous studies^89,90^. Although the effect of gender on civic role attitudes was not significant, according to social identity theory, males are more likely to consider farm animals potential food sources, while females may view farm animals as members of a social group, possibly resulting in female participants scoring higher than male in the citizen roles^16^.

Age was found not to be significantly associated with participants’ attitudes in either the consumer role or citizen role. This may be because the influence of age on attitudes is not direct but rather interacts with other factors, such as gender and ethical ideologies^15,16^. The results also revealed a potential nonlinear relationship between age and attitudes, where the attitude score initially decreased and then increased with age, showing a U-shaped curve. This pattern has also been observed in previous studies^91^. Furthermore, the consumer-citizen gap in attitudes was not statistically significant among participants aged 18–20 years. This observation may be attributed to the fact that individuals in this age group, typically junior college and university students, tend to exhibit a higher level of awareness and concern for animal protection^3,92^.

Education was found to have a significant and positive impact on attitudes in the dual role, which aligns with previous studies^10,13^. It is generally accepted that more educated individuals have greater access to information about farm animal welfare and are more aware of animal protection ^61^ In contrast to participants with other educational attainment, participants with postgraduate degrees displayed significantly more positive attitudes in the citizen role compared to the consumer role. This might be attributed to their altruistic values and their appreciation for the intrinsic value of animal life^68,93^.

Monthly household income represents the economic status of the public. Economically disadvantaged groups tend to view farm animals as similarly disadvantaged, and expressing greater care and sympathy for them^91^. Previous research findings also support this trend^34^. However, economically disadvantaged groups may face constraints in affording the premium of welfare-friendly animal products. As a result, among participants with monthly household incomes of less than US $1501, attitudes were significantly more positive in the citizen role than in the consumer role. In contrast, higher incomes provide greater purchasing power^94^, so participants with monthly household incomes exceeding US $1500 exhibited significantly more positive attitudes in the consumer role compared to the citizen role.

The results indicate that participants living in urban areas had more positive attitudes in the dual role, and the area of residence significantly influenced attitudes in the dual role. People living in urban areas, characterized by modern technological and industrialized lifestyles, tend to have a desire to return to nature and live in harmony with it^62^. This observation also helps explain the more positive attitudes in the citizen role among participants living in urban areas. Conversely, in the consumer role, the attitudes of participants living in rural areas were significantly more positive than those living in urban areas. This result is consistent with the previous study^95,96^. This may be because people in rural areas are accustomed to the traditional way of treating farm animals, and they are less informed about farm animal welfare or less inclined to support its improvement.

The results suggest that occupation did not significantly correlate with attitudes. However, when considering occupation, the attitudes of students were found to be the most positive. According to Roger’s theory of innovation diffusion, students generally possess characteristics such as diverse value orientations, active thinking, and strong innovative abilities, making them the primary audience group for farm animal welfare^97^. Conversely, participants who were farmers displayed the most negative attitudes. This aligns with the prevailing rural culture in China, where many farmers tend to view farm animals as natural resources that can be exploited rather than protected entities, regardless of whether animal husbandry is their primary occupation^98,99^. Unlike participants in other occupations, the consumer role attitude scores of those who are students and unemployed individuals are lower than the citizen role. This significant discrepancy may be attributed to their relatively limited experience in purchasing animal products or their financial constraints which prevent them from affording premium of welfare-friendly animal products^13^.

## Conclusions and limitations

In summary, this study draws some main conclusion. Public attitudes toward farm animal welfare in China are generally positive, with the majority of the public supporting improvements in farm animal welfare. The Chinese public has some distinctive characteristics related to farm animal welfare, including a conservative view of farm animal welfare, limited knowledge, and a tendency to free-ride. The current study reveals consumer-citizen gaps in Chinese public attitudes toward farm animal welfare. A significant consumer-citizen gap was found in overall attitudes, although the consumer role was only slightly more positive than the citizen role. This consumer-citizen gap is driven by differences in both cognitive attitudes and behavioral attitudes. The gap is most pronounced in cognitive attitudes, where the consumer role is significantly more positive, and smaller in behavioral attitudes, where the citizen role is significantly more positive. The consumer-citizen gap varies significantly among different demographic groups, including gender, age, education, monthly household income, area of residence, and occupation. Additionally, education, monthly household income, and area of residence have significant effects on attitudes in the dual role, whereas gender only affect consumer-role attitudes significantly.

However, there are several limitations to this study. First, the sample in this study may not perfectly reflect the distribution of the general Chinese population. Future research should aim to include a more diverse and representative sample. Second, while this study covered commonly examined demographic characteristics, it may not have accounted for all potential influencing factors. Future research should consider a wider range of influencing factors. Third, the text introducing the “Five Freedoms” concept appeared at the beginning of the questionnaire, which might have introduced bias due to socially desirable responses. Future research should seek ways to overcome this limitation. Finally, although the items in this study were not used to measure the consumer-citizen gap in previous studies, the method was supported by the theoretical methodology of previous studies. Nevertheless, this limitation cannot stop the development of such a tool as it would aid in the comprehension of the distinction between public attitudes toward farm animal welfare in their consumer and citizen roles.

## Data Availability

The datasets generated and/or analyzed during the current study are available from the corresponding author upon reasonable request.
